# The Mobilizable Plasmid P3 of Salmonella enterica Serovar Typhimurium SL1344 Depends on the P2 Plasmid for Conjugative Transfer into a Broad Range of Bacteria *In Vitro* and *In Vivo*

**DOI:** 10.1128/jb.00347-22

**Published:** 2022-11-16

**Authors:** Marla Sofie Gaissmaier, Leanid Laganenka, Mathias Klaus-Maria Herzog, Erik Bakkeren, Wolf-Dietrich Hardt

**Affiliations:** a Institute of Microbiology, ETH Zürich, Zürich, Switzerland; b Department of Biology, University of Oxford, Oxford, United Kingdom; c Department of Biochemistry, University of Oxford, Oxford, United Kingdom; University of Chicago

**Keywords:** antibiotic resistance, conjugation, infection biology

## Abstract

The global rise of drug-resistant bacteria is of great concern. Conjugative transfer of antibiotic resistance plasmids contributes to the emerging resistance crisis. Despite substantial progress in understanding the molecular basis of conjugation *in vitro*, the *in vivo* dynamics of intra- and interspecies conjugative plasmid transfer are much less understood. In this study, we focused on the streptomycin resistance-encoding mobilizable plasmid pRSF1010^SL1344^ (P3) of Salmonella enterica serovar Typhimurium strain SL1344. We show that P3 is mobilized by interacting with the conjugation machinery of the conjugative plasmid pCol1B9^SL1344^ (P2) of SL1344. Thereby, P3 can be transferred into a broad range of relevant environmental and clinical bacterial isolates *in vitro* and *in vivo*. Our data suggest that *S*. Typhimurium persisters in host tissues can serve as P3 reservoirs and foster transfer of both P2 and P3 once they reseed the gut lumen. This adds to our understanding of resistance plasmid transfer in ecologically relevant niches, including the mammalian gut.

**IMPORTANCE** *S.* Typhimurium is a globally abundant bacterial species that rapidly occupies new niches and survives unstable environmental conditions. As an enteric pathogen, *S.* Typhimurium interacts with a broad range of bacterial species residing in the mammalian gut. High abundance of bacteria in the gut lumen facilitates conjugation and spread of plasmid-carried antibiotic resistance genes. By studying the transfer dynamics of the P3 plasmid *in vitro* and *in vivo*, we illustrate the impact of *S.* Typhimurium-mediated antibiotic resistance spread via conjugation to relevant environmental and clinical bacterial isolates. Plasmids are among the most critical vehicles driving antibiotic resistance spread. Further understanding of the dynamics and drivers of antibiotic resistance transfer is needed to develop effective solutions for slowing down the emerging threat of multidrug-resistant bacterial pathogens.

## INTRODUCTION

Bacterial infections pose a high risk to human health. The use and overprescription of antibiotic treatments in human and veterinary medicine have been linked to the increasing emergence of antibiotic-resistant bacteria (1–3). According to the priority list published by the WHO concerning the increase of multidrug-resistant bacterial species, members of the *Enterobacteriaceae* family were classified as “most critical” (4). Furthermore, recent reports published by *The Lancet* claim that almost 5 million deaths were associated with antibiotic resistance in 2019 (5). Similarly, the spread of animal- or plant-associated pathogens with antibiotic resistance causes high treatment costs in agriculture and reduces the yield of certain crops (3, 6, 7). Understanding the mechanisms and slowing down the spread of antibiotic resistances of opportunistic or pathogenic bacteria are thus an important task to preserve antibiotics as an effective treatment strategy.

Horizontal gene transfer (HGT) is a dominant mechanism of acquiring antibiotic resistance (8). HGT mostly occurs by means of transformation, transduction, or conjugation (9). Out of these mechanisms, conjugation of plasmids is arguably the most important driver for spreading antibiotic resistance genes among bacteria (10). Plasmids are usually circular DNA molecules. In contrast to chromosomal DNA, they do not contain essential housekeeping genes but rather accessory genes that may be selected to improve the fitness of the host bacterium to adapt to particular environments—for example, by providing new metabolic functions, antibiotic resistance, or virulence (8, 11, 12). Generally, plasmids can be classified into three categories based on their mobility: (i) conjugative plasmids that encode their own conjugation machinery, (ii) mobilizable plasmids that do not encode a functional conjugation machinery, and (iii) nonmobilizable plasmids. It was estimated that conjugative and mobilizable plasmids make up around one-half of described plasmids (13).

In this study, we focused on the transfer dynamics of the mobilizable plasmid pRSF1010^SL1344^ (termed P3) (see Fig. S1 in the supplemental material), which naturally resides in Salmonella enterica serovar Typhimurium strain SL1344, an isolate from cattle that belongs to a clade of Salmonella strains contributing significantly to infections worldwide (14–16). P3 belongs to the IncQ incompatibility family of plasmids and is a close relative of the RSF1010 plasmid originally isolated from Escherichia coli (15, 17, 18). Similar to P3, it carries streptomycin (*strAB*) and sulfonamide resistance (*sulII*) genes that are typically contained within the same gene cluster and occur in various bacterial species (15, 19). *S*. Typhimurium strain SL1344 additionally harbors two conjugative plasmids, pSLT^SL1344^ and pCol1B9^SL1344^, which will be referred to as P1 and P2, respectively (20). *S*. Typhimurium typically contains 12 to 15 copies of P3, whereas only 1 to 2 copies of P1 and P2 are present per cell (17, 21, 22). In contrast to P3, which harbors only an origin of transfer (*oriT*) and genes required for mobilization (*mobABC*), the plasmids P1 and P2 encode complete conjugation machineries (17, 23, 24). P3 also encodes a plasmid-derived replication machinery (helicase, primase, and iteron-specific DNA-binding protein), allowing it to replicate in a host-independent manner (17, 25). Notably, the >99% similarity to the broad-host-range plasmid RSF1010 suggests that P3 might have a broad host range as well (see Fig. S1b in the supplemental material). For our study, this was of particular interest in combination with a host bacterium like *S*. Typhimurium that can grow both inside and outside the intestinal tract of host animals. Furthermore, this pathogen can form persister reservoirs (i.e., subpopulations of bacteria that survive upon exposure to antibiotics) in host tissues that might “store” plasmids over long periods of time (20, 26). Importantly, the persister populations can migrate back to the gut lumen after the antibiotic treatment has ended and resume gut-luminal growth (reseeding), further interacting with a significant number of bacterial species which inhabit or pass through the animal gut (27–29).

We show that P3 of *S*. Typhimurium can be mobilized by employing the conjugation machinery of the co-occurring P2 plasmid. Moreover, we found evidence for conjugational transfer of P3 to a variety of *Gammaproteobacteria* members not only *in vitro* but also in the animal gut. As *S*. Typhimurium persisters residing in host tissues can serve as P3 reservoirs and foster conjugative transfer of both P2 and P3 once they reseed the gut lumen, we speculate that the animal gut may be a relevant niche for spreading P3.

## RESULTS

### P3 requires P2 for conjugational transfer into a broad host range *in vitro*.

**FIG 1:**
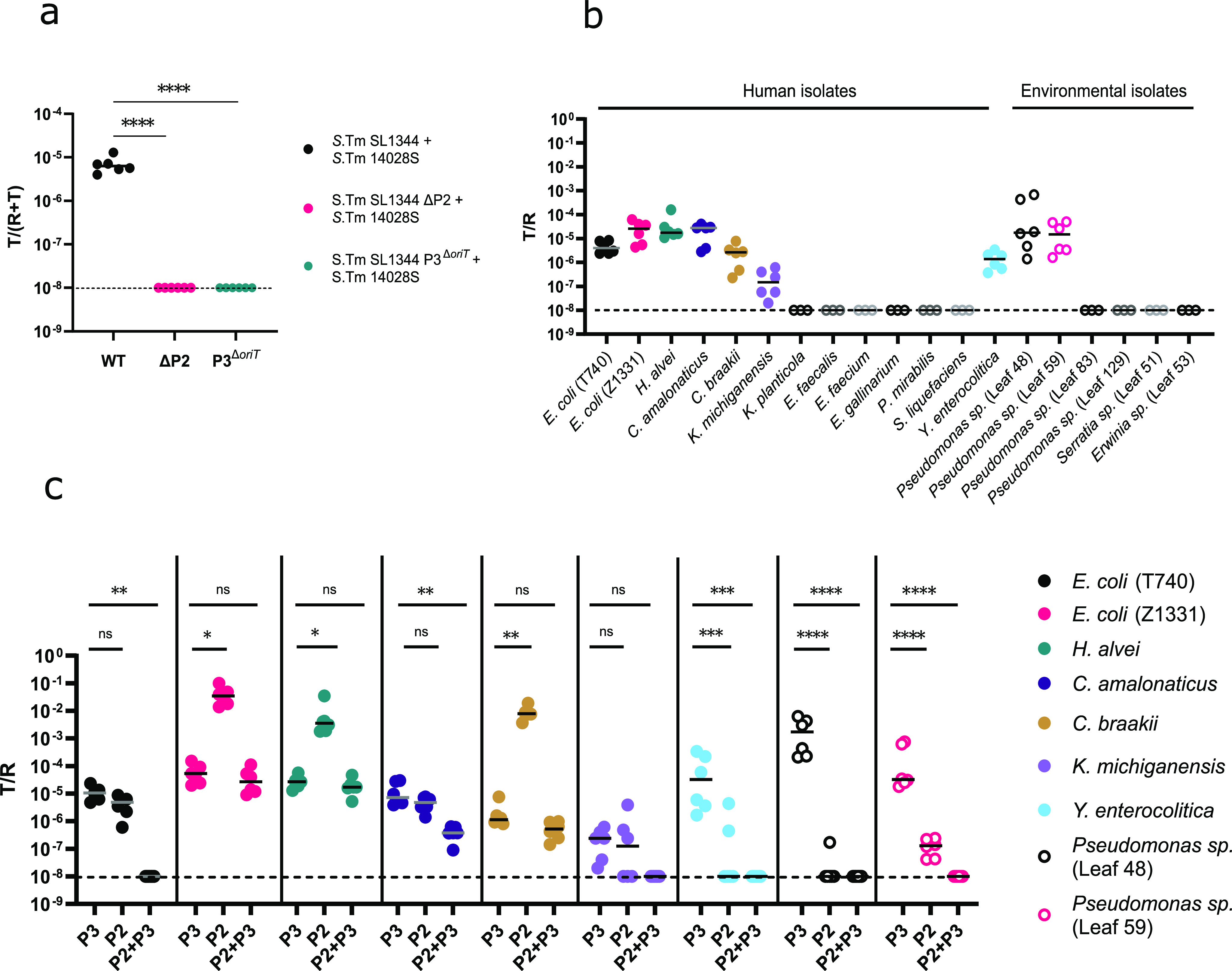
P3 is mobilized by P2 and can be stably maintained in a broad range of bacteria. (a) P2 is required for P3 transfer. Final conjugation frequencies (T/R+T ratio) of P3 for 2-h liquid mating of the three depicted donor/recipient combinations are shown. *S.* Typhimurium (*S*. Tm) SL1344 and 14028S recipient cells were mixed 1:1 in LB and incubated for 2 h. Respective plating on MacConkey plates was used for enumeration of donor, recipient, and transconjugant counts. *n* = 6 for each group, two independent experiments, *P* = 0.0022, two-tailed Mann-Whitney test. Limit of detection (LOD) = 10^−8^. (b) *In vitro* host range of P3. Shown is the ratio of transconjugants to recipients after overnight agar mating. A 1:1 mixture of the depicted recipient and *S.* Typhimurium SL1344 donor cells was incubated on LB agar overnight. *n* = 3 to 6 per group. LOD = 10^−8^. (c) Stable maintenance of P2 and P3 depends on the recipient strain. Shown is the T/R ratio of P3, P2, and P2 plus P3 transconjugants after overnight agar mating. A 1:1 mixture of recipient and *S.* Typhimurium 14028S/P2*^cat^* and -P3 cells was incubated on LB agar overnight. *n* = 6 per group, two independent experiments, one-way analysis of variance (ANOVA) and Bonferroni correction, comparing P3 with P2 or P3 with P2 plus P3. LOD = 10^−8^. *P* value significance: ns, 0.1 (not significant); *, 0.01; **, 0.001; ***, 0.0001; ****, 0.00001.

As P3 is transferred to other *S*. Typhimurium strains (Fig. 1a) but lacks the genes for a conjugation apparatus, we asked if conjugative plasmid P1 or P2 of *S*. Typhimurium SL1344 might facilitate conjugative P3 transfer. *S*. Typhimurium ATCC 14028S, which naturally lacks the plasmids P2 and P3, was used as a recipient strain, and transconjugants were detected after 2-h mating between SL1344 and 14028S in liquid medium (Fig. 1a). However, no transconjugants were observed when the donor strain was cured of P2. This suggests that P2 is required for transfer of the P3 plasmid. Disruption of the P3 origin of transfer gene, *oriT*, similarly abolished plasmid transfer, further confirming that it is indeed conjugation that drives the transfer of P3 (Fig. 1a).

To study the host range of the P3 plasmid, environmental and human bacterial isolates were selected based on the spectrum of antibiotic resistance (which should be different from that of the *S*. Typhimurium SL1344 donor strain) and ability to ferment lactose. This enabled detection and enumeration of P3 transconjugants based on differential plating or morphological differentiation of lactose-negative donor and lactose-positive recipient strains on MacConkey agar plates (see Fig. S2 in the supplemental material). The host range of P3 was investigated by performing overnight liquid and surface mating. For liquid mating, transconjugants could be detected in several human (Escherichia coli, Hafnia alvei, Citrobacter amalonaticus, and Yersinia enterocolitica) and environmental isolates (Pseudomonas sp. strains Leaf48 and Leaf59), albeit with different transconjugant yields (Fig. S3). P3 transfer was confirmed by PCR (Fig. S4a).

In contrast to previous work on other RSF1010 plasmids showing conjugative transfer to Gram-positive bacteria (30), no transconjugants were detected for Gram-positive representatives of the *Enterococcaceae* family (Enterococcus faecalis, Enterococcus faecium, and Enterococcus gallinarum) (Fig. 1b and Fig. S3). We speculate that this might be related to the specificity of the P2 sex pilus. Surface mating additionally enabled conjugative transfer of P3 into Citrobacter braakii and Klebsiella michiganensis, likely due to a closer contact between bacteria grown on a surface of an agar plate rather than in the liquid medium (Fig. 1b). The average ratio of detected CFU for transconjugants to recipients (T/R ratio) remained in a range of 10^−6^ to 10^−4^ for most tested strains, similar to the observed ratios in liquid mating. Although still above the detection limit, the T/R ratio of transconjugants for *K. michiganensis* was lower at around 5 × 10^−8^.

All strains that showed transconjugants were consequently mated with *S*. Typhimurium SL1344 P3*^ΔoriT^* to verify that plasmid uptake indeed occurred via conjugation and not by any other mechanism of horizontal gene transfer. No streptomycin-resistant CFU indicative of transconjugants were detected for any of the tested recipient strains when using an *oriT*-deficient P3, suggesting that conjugation was indeed the sole mechanism responsible for P3 transfer in all recipient strains (Fig. S4b).

### P2 and P3 are often cotransferred into the same recipients.

As our data above showed that P2 is required for P3 transfer, it was interesting to compare the P2 host range and conjugation rate to those of P3. We chose *S*. Typhimurium 14028S harboring P2*^cat^* and P3 as the donor for all mating pairs, allowing us to follow the dynamics of P2 transfer by differential plating using chloramphenicol as a selection marker. After overnight agar mating, P2*^cat^* was detected in high numbers for the tested E. coli and *H. alvei* strains. Both tested environmental samples (Pseudomonas sp. strains Leaf48 and Leaf59) as well as *K. michiganensis* and Y. enterocolitica showed low or undetectable levels of P2*^cat^* transconjugants (Fig. 1c). These findings suggest that although P3 stably replicates upon transfer into a recipient strain, P2 seems to be mostly lost from the recipient cells of Pseudomonas sp. strains Leaf48 and Leaf59, *K. michiganensis*, and Y. enterocolitica within 24 h. With P2 being a large and low-copy plasmid, possible reasons for plasmid loss could be high fitness burden on the host or segregational loss (31). We additionally analyzed the genomes of the recipient strains for plasmids of the same incompatibility complex as that of P2 (Incl1). No potentially incompatible plasmids were detected in the strains based on our BLAST analysis of the conserved replication initiation protein RepZ of IncI1 plasmids (32). Interestingly, subsequent surface mating of all P3 transconjugants with a naive recipient E. coli strain (W3110 *tsr*::Km^r^) further confirmed that conjugative P3 transfer was only possible for donor cells inheriting both P2 and P3 plasmids (Fig. S4c).

### P3 transfer *in vivo*.

**FIG 2:**
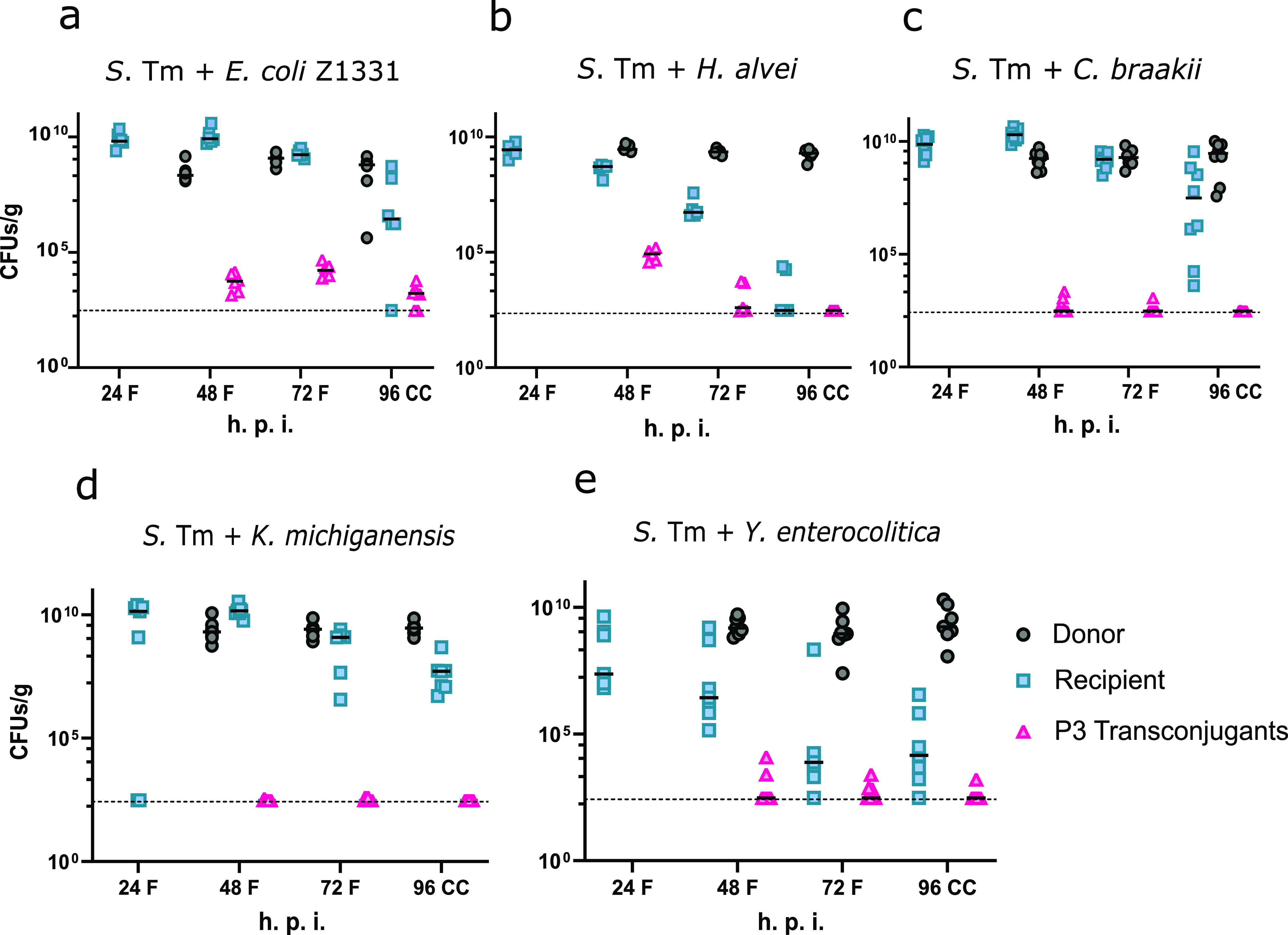
P3 transfer occurs in the gut and is not limited to one species. Shown are total CFU counts of donor, recipient, and transconjugant populations in the feces (F) and cecal contents (CC). Ampicillin-pretreated C57BL/6 mice were infected with 5 × 10^7^ CFU of the recipient strain on day 0 and with 5 × 10^7^ CFU of the donor strain *S*. Typhimurium SL1344 on day 1. The tested recipients are (a) E. coli Z1331 WITS1 Amp^r^ *lac*^+^ (*n* = 6) (b) *H. alvei*/pM965 (Amp^r^)/pACYC184 (Cm^r^) (*n* = 5), (d) *K. michiganensis* (Amp^r^ *lac*^+^) (*n* = 5), (c) *C. braakii*/pM965 (Amp^r^ *lac*^+^) (*n* = 8), (e) Y. enterocolitica/pM965 (Amp^r^ Km^r^) (*n* = 7). Donors, recipients, and transconjugants are indicated as gray circles, cyan squares, and pink triangles, respectively. h.p.i., hours postinfection.

The main ecological niche occupied by the P3 plasmid-carrying *S*. Typhimurium is the mammalian gut. We therefore used a well-established mouse colitis model (20, 24, 33) to verify the previous *in vitro* results and to study the dynamics of P3 transfer in this niche. C57BL/6 specific-pathogen-free (SPF) mice were pretreated with ampicillin to suppress the microbiota and allow the strains of interest to grow up to high densities, which was necessary for successful conjugation (24). The mice were then infected with respective recipient strains 24 h prior to infection with the *S*. Typhimurium SL1344 donor strain. The number of transconjugants was determined by daily collection and differential plating of feces and cecal content for the next 3 days. Transconjugants were detected in E. coli Z1331, *H. alvei*, *C. braakii*, and Y. enterocolitica (Fig. 2a, b, c, and e, respectively). No transconjugants were detected in *K. michiganensis* (Fig. 2d). This shows that P3 indeed gets transferred between species within the gut system if donor and/or recipient strains can grow up to high densities.

### Reseeding of *S*. Typhimurium persisters as a P3 reservoir.

*S*. Typhimurium not only can invade the gut tissue but also colonizes systemic organs and forms long-term reservoirs at these sites (34–36). From these reservoirs, the pathogen can again pass the epithelial barrier and thereby reseed into the gut lumen. 129S6/SvEvTac mice were used to analyze the role of reseeding *S*. Typhimurium persisters as a P3 reservoir. Compared to C57BL/6 mice, the 129S6/SvEvTac mouse line is more resistant to *S*. Typhimurium infection, allowing for long-term infections (37).

**FIG 3:**
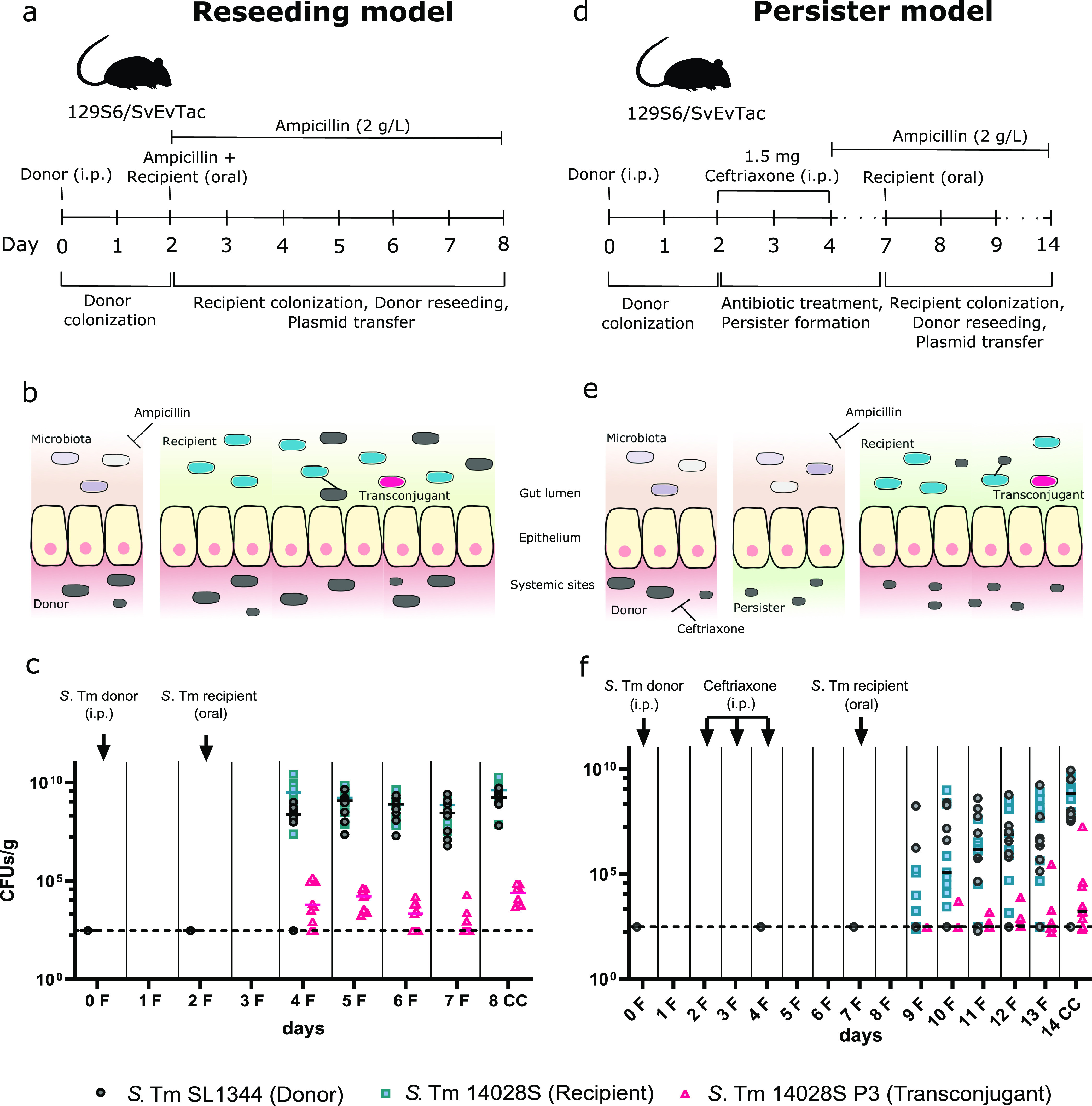
Systemic infection with *S.* Typhimurium (*S*. Tm) SL1344 shows conjugative P3 transfer after donor reseeding from tissue reservoirs into the gut lumen regardless of antibiotic treatment. (a) Experimental setup for reseeding donor model adapted from Bakkeren et al. (20, 26). The reseeding donor model contained two phases: phase 1, donor colonization and establishment of tissue reservoirs at systemic sites; phase 2, recipient colonization in the gut lumen, donor reseeding, and plasmid transfer via conjugation. (b) Reseeding model. *S.* Typhimurium donors (gray) establish tissue reservoirs after intraperitoneal injections and spread to organs. The microbiota provides colonization resistance against donor colonization of the gut lumen. Recipients (cyan) colonize the gut lumen by an oral infection following an ampicillin treatment to suppress the microbiota. (The presence of antibiotics is indicated by green shading.) *S.* Typhimurium donors reseed from their reservoirs with subsequent plasmid transfer to recipients (indicated by the line between them) and formation of transconjugants (pink). (c) Conjugative P3 transfer by reseeding donors from tissue reservoirs into the gut lumen. Mice were infected with 5 × 10^3^ CFU of the donor *S.* Typhimurium SL1344 (Amp^r^ Km^r^ Sm^r^) on day 0 (i.p.) and with 5 × 10^7^ CFU of the recipient *S.* Typhimurium 14028S (Amp^r^ Cm^r^) on day 2 (oral), 4 h post-oral ampicillin pretreatment (20 mg). Ampicillin was added at 2 g/L to the drinking water on day 2 postinfection and maintained throughout the experiment. CFU counts of donor, recipient, and transconjugant populations in the feces and cecal contents were determined by differential plating. Two mice were excluded from the analysis as they developed severe symptoms and were euthanized earlier for ethical reasons. *S.* Typhimurium donor was invariably detected in the systemic sites (Fig. S6a). *n* = 8, two independent experiments. (d) Experimental setup for the reseeding persister model adapted from Bakkeren et al. (20, 26) The reseeding persister model contained three phases: phase 1, donor colonization and establishment of tissue reservoirs at systemic sites; phase 2, clearance with antibiotics and persister formation at systemic sites; phase 3, recipient colonization in the gut lumen, donor reseeding, and plasmid transfer via conjugation. (e) Persister model. *S.* Typhimurium donors (gray) establish tissue reservoirs after intraperitoneal injections and spread to organs. The microbiota provides colonization resistance against donor colonization of the gut lumen. Upon ceftriaxone treatment, only persisting *S.* Typhimurium cells can survive, which leads to new tissue reservoir formation. (The presence of antibiotics is indicated by green shading.) Recipients (cyan) colonize the gut lumen by oral infection following an ampicillin treatment (supplemented in the water) to suppress the microbiota. *S.* Typhimurium donors reseed from their newly established reservoirs with subsequent plasmid transfer to recipients and transconjugant formation (pink) (20). (f) Conjugative P3 transfer by reseeding persisters from tissue reservoirs into the gut lumen. Mice were infected with 5 × 10^7^ CFU of the donor *S.* Typhimurium SL1344 (Amp^r^ Km^r^ Sm^r^) on day 0 (i.p.) and with 5 × 10^3^ CFU of the recipient *S.* Typhimurium 14028S (Amp^r^ Cm^r^) on day 7 (oral). Ampicillin was added at 2 g/L to the drinking water on day 4 and maintained throughout the experiment to suppress the gut microbiota for subsequent oral infection with the recipient strain. CFU counts of donor, recipient, and transconjugant populations in the feces and cecal contents were determined by differential plating. One mouse had to be excluded from the analysis as it developed severe symptoms on day 10 and was euthanized for ethical reasons. The *S.* Typhimurium donor was invariably detected in the systemic sites (Fig. S6b). *n* = 9, two independent experiments. F, fecal sample; CC, cecal content.

To maintain a donor-free gut lumen and establish systemic *S*. Typhimurium infection at the same time, mice were infected with the SL1344 donor strain (*S*. Typhimurium SL1344/P3^SmR^/pM965^ApR^) by intraperitoneal (i.p.) injection. Two days postinfection (dpi), the mice were orally treated with ampicillin to suppress the microbiota and subsequently infected with the 14028S recipient strain (*S*. Typhimurium ATCC 14028S *marT*::*cat* Cm^r^) by oral gavage. The dynamics of donor reseeding and subsequent conjugation were quantified for the following 6 days (Fig. 3a and b). Donor reseeding occurred typically within the first 3 days post-antibiotic treatment (Fig. 3c and Fig. S6a). P3 transconjugants were detected in all tested mice as soon as reseeding had occurred. No transconjugants could be detected when *S*. Typhimurium SL1344/P3^Δ^*^oriT^* was used as the plasmid donor, confirming that conjugation is the only route for P3 transfer in this model (Fig. S5).

We further assessed the transfer dynamics of P3 for persisting *S*. Typhimurium donors. *S*. Typhimurium persisters residing at systemic sites can subsequently reenter the gut lumen (38) and can serve as a P2 reservoir (20). These tissue-lodged persister reservoirs are of special concern as they survive antibiotic treatments for a longer period of time and can lead to recurring or chronic infections (39). Because our previous *in vitro* experiments implied that P2 mobilizes P3, we did a follow-up experiment to detect whether P3 would be transferred along with P2 if both derive from persistent *S*. Typhimurium donors. To focus our analysis on the plasmids that are carried by tissue-lodged persisters, we extended the reseeding model described above by a 3-day i.p. ceftriaxone treatment to eliminate all non-tissue-lodged *S*. Typhimurium cells. The drinking water was supplemented with ampicillin to suppress the gut microbiota and open a niche for the recipient (Fig. 3d and e). The 14028S recipient strain was orally introduced on day 7. The dynamics of donor reseeding and subsequent conjugation were quantified for the following 7 days.

Persister reseeding as well as P3 transfer to the recipient strain were detected in 7 out of 9 mice by 14 dpi (Fig. 3f and Fig. S6b). The lower counts of P3 transconjugants in this experimental setup (Fig. 3d to f) than in the reseeding model (Fig. 3a to c) could be attributed to lower gut-luminal densities of both donor and recipient strains and higher variation in CFU between the animals. Nevertheless, our data clearly show that *S*. Typhimurium persisters serve as an antibiotic resistance plasmid reservoir, which leads to subsequent gut-luminal transfer not only of conjugative plasmids such as P2, as shown in previous studies (20), but also of mobilizable plasmids such as P3, as shown here.

## DISCUSSION

Conjugative plasmid transfer is a critical driver of antibiotic resistance spread among bacteria (40–42). Investigation of the distribution and transfer dynamics of broad-host-range plasmids is of particular importance, as they can potentially contribute to HGT between distantly related bacterial species. This study focused on the host range and transfer dynamics of the mobilizable plasmid P3 (IncQ family) of *S*. Typhimurium SL1344 *in vitro* and *in vivo*. As transfer of mobilizable plasmids is dependent on external genes that encode the conjugation machinery, we aimed at determining the source of the conjugation machinery explaining P3 transfer. Our data suggest that the conjugative P2 plasmid of *S*. Typhimurium SL1344 is responsible for P3 transmission. P3 transfer was additionally abolished by deletion of its origin of transfer (*oriT*), further confirming that it is indeed conjugation and no other type of HGT that drives P3 transfer. Since we have not tested a P1-deficient *S*. Typhimurium donor strain, no final assumptions about its importance for P3 mobilization can be made. However, it is known that P1 is less efficiently transferred via HGT than P2 and that SL1344 donors harboring only P1 were not able to transfer P3 into the recipient 14028S strain (23, 43).

Because of the ability to replicate in a variety of bacteria, broad-host-range plasmids are of special concern if they harbor antibiotic resistance genes (44). Plasmids of the IncQ family are usually associated with streptomycin and sulfonamide resistance, but additional clinically relevant antibiotic resistance genes have been reported (17, 45). In line with previous studies that showed the broad host range of RSF1010-like plasmids (17, 46, 47), we could detect P3 transfer into a range of human isolates. Interestingly, P3 transconjugants were also detected in two environmental isolates of Pseudomonas (Leaf48 and Leaf59). Although P3 may thus promote rapid spread of streptomycin resistance in both commensal and pathogenic plant-associated bacteria, our study implies that the spread of P3 in a given population is limited by the efficiency of P2 transfer and maintenance within the recipient cell. Our data suggest that the emergence of P3 transconjugants does not necessarily coincide with successful P2 plasmid transfer or maintenance. In this case, if the recipient cell does not harbor another plasmid that could be exploited by P3 for further conjugation, no further spread of antibiotic resistance by HGT is possible. The reasons behind the differential ability of transconjugants to stably maintain the P2 plasmid are yet to be studied. In conclusion, our *in vitro* experiments show the dynamics of P3 plasmid transfer and how P3 can take advantage of the conjugative plasmid P2 to enter new host cells.

Our *in vivo* studies further verify that the animal gut is a niche permitting resistance plasmid transfer. The data extends previous work by showing P3 transfer into *H. alvei*, *C. braakii*, and Y. enterocolitica recipients. Additionally, we established that tissue-resident *S*. Typhimurium cells can serve as P3 donors after reseeding the gut lumen. The transfer occurred independently of selective pressure for the streptomycin resistance encoded on P3, and the P3 transconjugant population was stably maintained at a level of up to 10^5^ CFU/g feces or cecal content for mice with reseeding donors throughout the experiment.

Persisters residing at systemic sites are of special concern as they can survive antibiotic treatments over long periods of time and can lead to reoccurring infections by reseeding into the gut lumen (48). Our data further demonstrate the possibility of P3 transfer from persister reservoirs. Its ability to spread antibiotic resistance-carrying broad-host-range plasmids like P3 renders *S*. Typhimurium a concerning driver of antibiotic resistance spread on a global scale.

*S*. Typhimurium might be of particular importance in driving conjugative HGT for two reasons. Not only can it form tissue reservoirs, including persisters (as discussed above), but it also causes severe inflammation and thus leads to dysbiosis, enabling blooms of potential recipient cells (especially members of the *Proteobacteria* [49]) and thereby promoting conjugation (24). In general, this further confirms how dysbiosis (which can be induced by many factors like diet, stress, antibiotic treatment, or diseases [50–53]) might boost HGT and antibiotic resistance spread as soon as a potent donor strain like *S*. Typhimurium SL1344 enters the gut system (24). As P2 is a close relative to the clinically relevant extended-spectrum β-lactamase (ESBL) plasmids (26), it might be interesting to investigate how well ESBL plasmids are able to mobilize P3 or closely related plasmids from the IncQ family. Overall, the presented data add to our understanding of resistance plasmid transfer and suggest that conjugation and plasmid maintenance in gut luminal microbes and environmental bacteria should be considered in health approaches aiming to decipher and reduce the spread of antibiotic resistance.

## MATERIALS AND METHODS

### Bacterial strains and growth conditions.

The strains and plasmids used in this study are listed in Tables S1 and S2 in the supplemental material. All cells were routinely grown on either 1.5% lysogeny broth (LB) agar, MacConkey agar, or liquid LB supplemented with ampicillin (100 μg/mL), kanamycin (50 μg/mL), streptomycin (50 μg/mL), or chloramphenicol (35 μg/mL) where necessary. Gene deletions were obtained via PCR-based inactivation (54).

### Recipient screen.

To study the host range of the P3 plasmid, bacterial strains from environmental and human isolates were screened for their spectrum of antibiotic resistance and the ability to ferment lactose to enable later differentiation from the *S*. Typhimurium donor strains on MacConkey agar plates.

To detect antibiotic resistance for all potential recipient strains, a MIC assay was conducted for ampicillin, streptomycin, chloramphenicol, and kanamycin. Twenty-four-well plates (TPP; no. 92024) were filled with 1 mL LB medium and supplemented with a range of concentrations of the respective antibiotics from 0 to 100 μg/mL. Tested strains were grown in 5 mL LB overnight at 30 or 37°C. The optical density at 600 nm (OD_600_) of the cultures was adjusted to 1.0. Subsequently, 10 μL of the culture was added to each well. The plates were incubated at 30°C or 37°C overnight, and OD_600_ was measured for each well.

### *In vitro* mating.

Two-hour liquid mating was performed to determine conjugation frequencies. The donor and recipient strains were grown individually, and after a washing step to ensure the removal of antibiotics, 10 μL (~1 × 10^7^ CFU) of both donor and recipient cells was added to 1 mL LB. The cells were incubated at 37°C for 2 h without shaking. To determine the numbers of donors, recipients, and transconjugants, dilution series were plated on MacConkey or LB agar (depending on the recipient strain) supplemented with respective antibiotics. The conjugation frequency (CF) was determined by the formula in equation 1:

(1) $$\text{CF = }\frac{\text{Transconjugants (CFU/mL) }}{\text{Transconjugants (CFU/mL) }+\text{ Recipients (CFU/mL) }}$$

To determine whether P3 was expressed stably in a recipient bacterium, the same experiment was conducted for a longer period. Twenty microliters of the 1:1 mixture of donor and recipient cells was added to 5 mL of LB medium without antibiotics and incubated overnight without shaking. The numbers of donors, recipients, and transconjugants were determined by respective plating. The ratio between transconjugant counts and recipient counts (T/R ratio) was used to normalize the data set in order to compare different recipient strains (equation 2):

(2) $$\text{T/R ratio = }\frac{\text{Transconjugants (CFU/mL) }}{\text{Recipients (CFU/mL) }}$$

Mating experiments on agar as a solid surface were added to this study as conjugation events require close physical contact of recipient and donor cells (5). Agar mating enabled an additional way of investigating the host range of P3 because it allowed higher densities of recipient and donor strains at the same spot than liquid mating. The donor and recipient strains were cultured and prepared as described above, and 10-μL droplets of a 1:1 mixture were pipetted onto LB agar without antibiotics. After incubation overnight, the cultures were scraped off the agar and resuspended in 1 mL of 1× phosphate-buffered saline (PBS). Donors, recipients, and transconjugants were enumerated as described above.

### Isolation of human *Enterobacteriaceae* from stool samples.

The feces examined in this study originated from healthy donors and from salmonellosis patients in remission of two different clinical trials. The feces were collected and homogenized with tryptic soy broth plus glycerol before being stored at −80°C. For plating, an aliquot of the sample was scraped off the frozen stock and homogenized for 1 min at 25 Hz in PBS using a Qiagen TissueLyser. Serial dilutions were prepared in PBS and plated on MacConkey agar (Oxoid; CM0007) (with incubation overnight at 37°C under aerobic conditions). Morphologically different colonies were picked and streaked on MacConkey agar to ensure purity of the culture (with incubation overnight at 37°C under aerobic conditions). A single colony was picked to inoculate a 3-mL liquid LB culture (37°C overnight). The pellet of the culture was used for DNA extraction (Qiagen DNeasy blood and tissue kit).

### DNA extraction and sequencing.

The Qiagen DNeasy blood and tissue kit protocol was used, and the DNA was eluted in 50 μL elution buffer (AE). The DNA samples were sent to Novogene for library preparation, sequencing (Illumina NovaSeq 6000), and raw read filtering (BioProject accession no. PRJNA853708).

### Bioinformatics.

**(i) Identification of the species.** The paired-end reads were *de novo* assembled with CLC Workbench 20.0.4. Contigs with lengths below 500 bp were discarded, and the species of the isolates was identified by multilocus sequence typing (55). The assemblies of the Leaf isolates (56) (Fig. S1) were downloaded from GenBank (NCBI, BioProject accession no. PRJNA224116 and PRJNA297956).

**(ii) Search for *repZ*.** The sequence of the *repZ* gene was downloaded from NCBI, extracted from *S*. Typhimurium SL1344 (accession no. HE654725.1) (15), and used for two sorts of BLAST (57) searches (megablast and blastn) on the contigs/assemblies.

### Mouse models.

Short-term experiments (6 days) were carried out with 8- to 12-week-old, specific-pathogen-free (SPF) C57BL/6 mice (JAX:000664; The Jackson Laboratory). Long-term experiments (9 to 15 days) were carried out with 8- to 12-week-old SPF 129S6/SvEvTac mice (RRID:IMSR_TAC:129sve). All mice were bred at the EPIC mouse facility at ETH Zürich. During the experiment, they were housed under barrier conditions in individually ventilated cages at the ETH Zürich rodent center (RCHCI) with a maximum of 5 mice per cage. The mice were fed with an autoclaved mouse maintenance diet (Kliba Nafag; 3537) (by weight containing 4.5% fat, 18.5% protein, ~50% carbohydrates, and 4.5% fiber). Mice of both sexes were randomly assigned to experimental groups. All infection experiments were approved by the responsible authorities (Tierversuchskommission Kantonales Veterinäramt Zürich, license ZH158/2019).

**(i) Model for interspecific conjugation.** Ampicillin-pretreated 8- to 12-week-old SPF C57BL/6 mice were orally infected by gavaging 5 × 10^7^ CFU of the recipient strain 1 day prior to oral infection with 5 × 10^7^ CFU of the donor strain. To prepare the inoculum, the relevant strains were cultured in LB with selective antibiotics overnight and subcultured (1:20 dilution) until they reached an OD_600_ of 0.5 to 1.0 (exponential growth phase) and then adjusted to an OD of 1.0 by being washed and diluted in sterile 1× PBS. The transfer of P3 was monitored for a total of 3 days by respective plating of fecal samples for the donor, recipient, and transconjugant populations on MacConkey or LB agar (depending on the recipient strain). Fecal samples were collected every 24 h and placed in preweighed (weighed before [wb]) 2-mL Eppendorf tubes which contained 500 μL of sterile 1× PBS and a metal bead. The tubes were weighed again (wa) after sample collection, and the samples were homogenized for 2 min at 25 Hz using a TissueLyser from Qiagen. At 4 dpi, the mice were sacrificed by CO_2_ asphyxiation. Cecal content as well as mesenteric lymph nodes (mLNs), liver, and spleen were collected and plated. The number of cells per organ was calculated by multiplying the counted CFU by a factor of 10 for lymph nodes and spleen and by a factor of 60 for the liver as only 1/6 of it was collected and plated. The formula in equation 3 was used to determine the number of CFU per gram of feces or cecal content:

(3) $$\frac{\text{CFU}}{\text{g}}\text{= }\frac{\text{CFU × Dilution}}{\text{wa}-\text{wb}}$$

**(ii) Reseeding donor model.** *S*. Typhimurium can invade host cells during a gastrointestinal infection and colonize systemic organs. Its ability to pass the epithelial barrier within the gut system by employing a type III secretion system can lead to reseeding events into the gut lumen (20, 58, 59). The described model was adapted from previous studies (20, 26). Ampicillin-pretreated 8- to 12-week-old SPF 129S6/SvEvTac mice were chosen to investigate the intraspecific transfer of P3 by a reseeding *S*. Typhimurium donor for a total of 8 days. In contrast to C57BL/6, this mouse line has a functional allele for Nramp1 (also known as Slc11a1). The encoded solute carrier protein is a host resistance factor and allows for long-term infections with *S*. Typhimurium as it restricts Salmonella growth through deprivation of the crucial micronutrient Mn^2+^ (37, 60). The experiment contained two phases: phase 1, days 0 to 2, donor colonization; phase 2, days 2 to 8, recipient colonization, donor reseeding, and plasmid transfer. In phase 1, mice were i.p. infected with 10^3^ CFU of the donor strain *S*. Typhimurium SL1344 WITS1 Km^r^/pM965 on day 0 (subcultured for 2.5 h and with OD_600_ adjusted to 1.0). In phase 2, mice were pretreated with 20 mg ampicillin on day 2 and 4 h later infected by oral gavage with 5 × 10^7^ CFU of the recipient strain *S*. Typhimurium 14028S *marT*::*cat*/pM965 (subcultured for 2.5 h and with OD_600_ adjusted as described above). Sterile-filtered ampicillin (2 g/L) was added to the drinking water on day 2 after infection and maintained throughout the experiment to ensure continuous suppression of microbiota growth and stable colonization of donor and recipient strains. The transfer of P3 was monitored for a total of 5 days (days 4 to 8) by respective plating of fecal samples (days 4 to 7), and cecal content, mesenteric lymph nodes (mLNs), liver, and spleen (day 8) for donor, recipient, and transconjugant cells. The mice were housed in individual cages throughout the experiment to prevent cross-contamination and minimize the risk of donor infections via the fecal-oral route and were euthanized by CO_2_ asphyxiation 8 dpi. The entire experimental setup is illustrated in Fig. 3a and b.

**(iii) Persisting donor model.** The experiment consisted of three phases: phase 1, days 0 to 2, donor colonization; phase 2, days 2 to 7, antibiotic clearance; phase 3, days 7 to 14, recipient colonization, reseeding of the persisting donors from tissue reservoirs, and conjugative plasmid transfer. In phase 1, mice were i.p. infected with the donor (10^3^ CFU) on day 0. In phase 2, on days 3, 4, and 5 the mice were treated with 1.5 mg ceftriaxone intraperitoneally to induce persister formation of the donor strain. The mice were put in separate cages after the last ceftriaxone treatment to minimize donor infections via coprophagy and cross-contamination. Additionally, ampicillin (2 g/L) was added to the drinking water on day 4 and maintained throughout the experiment to suppress the microbiota for a later introduction of the recipient strain *S*. Typhimurium 14028S *marT::cat*/pM965. In phase 3, the recipient was introduced orally on day 7 by gavaging 5 × 10^7^ CFU. The inoculum was prepared as mentioned above. Feces were collected and plated on MacConkey agar with respective antibiotics on days 0, 2, and 7 to check for (unwanted) donor colonization in the gut and on days 9 to 14 to enumerate donor, recipient, and transconjugant populations. The mice were euthanized at 14 dpi by CO_2_ asphyxiation, and cecal content, mNLs, liver, and spleen were collected and plated as described above. The experimental setup can be looked up in Fig. 3d and e.

### Data analysis.

Microsoft Excel 2016 for Windows was used to calculate the number of cells per gram for all *in vivo* experiments. Data were analyzed and plotted using GraphPad Prism version 9.2.0 for Windows (GraphPad Software, La Jolla, CA, USA [www.graphpad.com]). For statistical analysis, the significance level was set to 5%.

### Data availability.

All raw data and executed protocols gathered during this study are available upon request from Wolf-Dietrich Hardt. Genomic DNA sequences (raw sequence reads) of *K. michiganensis* T737, E. faecalis T749, *C. amalonaticus* T747, P. mirabilis T746, E. coli T740, *E. gallinarum* T727, E. faecium T707, *C. braakii* T706, and *H. alvei* Z6026 are accessible from NCBI under BioProject accession no. PRJNA853708.

## References

[1] Clatworthy AE, Pierson E, Hung DT. 2007. Targeting virulence: a new paradigm for antimicrobial therapy. Nat Chem Biol 3:541–548. 10.1038/nchembio.2007.24. 17710100

[2] Van Boeckel TP, Brower C, Gilbert M, Grenfell BT, Levin SA, Robinson TP, Teillant A, Laxminarayan R. 2015. Global trends in antimicrobial use in food animals. Proc Natl Acad Sci USA 112:5649–5654. 10.1073/pnas.1503141112. 25792457 PMC4426470

[3] McManus PS, Stockwell VO, Sundin GW, Jones AL. 2002. Antibiotic use in plant agriculture. Annu Rev Phytopathol 40:443–465. 10.1146/annurev.phyto.40.120301.093927. 12147767

[4] WHO. 2017. WHO publishes list of bacteria for which new antibiotics are urgently needed. World Health Organization, Media Centre. https://www.who.int/news/item/27-02-2017-who-publishes-list-of-bacteria-for-which-new-antibiotics-are-urgently-needed.

[5] Murray CJ, Ikuta KS, Sharara F, Swetschinski L, Robles Aguilar G, Gray A, Han C, Bisignano C, Rao P, Wool E, Johnson SC, Browne AJ, Chipeta MG, Fell F, Hackett S, Haines-Woodhouse G, Kashef Hamadani BH, Kumaran EAP, McManigal B, Agarwal R, Akech S, Albertson S, Amuasi J, Andrews J, Aravkin A, Ashley E, Bailey F, Baker S, Basnyat B, Bekker A, Bender R, Bethou A, Bielicki J, Boonkasidecha S, Bukosia J, Carvalheiro C, Castañeda-Orjuela C, Chansamouth V, Chaurasia S, Chiurchiù S, Chowdhury F, Cook AJ, Cooper B, Cressey TR, Criollo-Mora E, Cunningham M, Darboe S, Day NPJ, De Luca M, Dokova K, et al. 2022. Global burden of bacterial antimicrobial resistance in 2019: a systematic analysis. Lancet 399:629–655. 10.1016/S0140-6736(21)02724-0. 35065702 PMC8841637

[6] Stockwell VO, Duffy B. 2012. Use of antibiotics in plant agriculture. Rev Sci Tech 31:199–210. 10.20506/rst.31.1.2104. 22849276

[7] He Y, Yuan Q, Mathieu J, Stadler L, Senehi N, Sun R, Alvarez PJJ. 2020. Antibiotic resistance genes from livestock waste: occurrence, dissemination, and treatment. NPJ Clean Water 10.1038/s41545-020-0051-0.

[8] Ochman H, Lawrence JG, Groisman EA. 2000. Lateral gene transfer and the nature of bacterial innovation. Nature 405:299–304. 10.1038/35012500. 10830951

[9] Brito IL. 2021. Examining horizontal gene transfer in microbial communities. Nat Rev Microbiol 19:442–453. 10.1038/s41579-021-00534-7. 33846600

[10] Wang Y, Batra A, Schulenburg H, Dagan T. 2022. Gene sharing among plasmids and chromosomes reveals barriers for antibiotic resistance gene transfer. Philos Trans R Soc Lond B Biol Sci 377:20200467. 10.1098/rstb.2020.0467. 34839702 PMC8628082

[11] Summers DK. 1996. The biology of plasmids. Blackwell Science, Ltd, Oxford, United Kingdom.

[12] Eberhard WG. 1989. Why do bacterial plasmids carry some genes and not others? Plasmid 21:167–174. 10.1016/0147-619X(89)90040-1. 2675150

[13] Coluzzi C, Garcillan-Barcia MP, de la Cruz F, Rocha EPC. 2022. Evolution of plasmid mobility: origin and fate of conjugative and nonconjugative plasmids. Mol Biol Evol 39:msac115. 10.1093/molbev/msac115. 35639760 PMC9185392

[14] Kingsley RA, Bäumler J. 2000. Host adaptation and the emergence of infectious disease—the Salmonella paradigm. Mol Microbiol 36:1006–1014. 10.1046/j.1365-2958.2000.01907.x. 10844686

[15] Kröger C, Dillon SC, Cameron ADS, Papenfort K, Sivasankaran SK, Hokamp K, Chao Y, Sittka A, Hébrard M, Händler K, Colgan A, Leekitcharoenphon P, Langridge GC, Lohan AJ, Loftus B, Lucchini S, Ussery DW, Dorman CJ, Thomson NR, Vogel J, Hinton JCD. 2012. The transcriptional landscape and small RNAs of *Salmonella enterica* serovar Typhimurium. Proc Natl Acad Sci USA 109:E1277–E1286. 22538806 10.1073/pnas.1201061109 PMC3356629

[16] Rankin JD, Taylor RJ. 1966. The estimation of doses of *Salmonella* typhimurium suitable for the experimental production of disease in calves. Vet Rec 78:706–707. 5336163 10.1136/vr.78.21.706

[17] Rawlings DE, Tietze E. 2001. Comparative biology of IncQ and IncQ-like plasmids. Microbiol Mol Biol Rev 65:481–496. 10.1128/MMBR.65.4.481-496.2001. 11729261 PMC99038

[18] Guerry P, van Embden J, Falkow S. 1974. Molecular nature of two nonconjugative plasmids carrying drug resistance genes. J Bacteriol 117:619–630. 10.1128/jb.117.2.619-630.1974. 4590480 PMC285553

[19] Yau S, Liu X, Djordjevic SP, Hall RM. 2010. RSF1010-like plasmids in Australian *Salmonella enterica* serovar Typhimurium and origin of their sul2-strA-strB antibiotic resistance gene cluster. Microb Drug Resist 16:249–252. 10.1089/mdr.2010.0033. 20617928

[20] Bakkeren E, Huisman JS, Fattinger SA, Hausmann A, Furter M, Egli A, Slack E, Sellin ME, Bonhoeffer S, Regoes RR, Diard M, Hardt W-D. 2019. *Salmonella* persisters promote the spread of antibiotic resistance plasmids in the gut. Nature 573:276–280. 10.1038/s41586-019-1521-8. 31485077 PMC6744281

[21] Sanchez-Romero MA, Merida-Floriano A, Casadesus J. 2020. Copy number heterogeneity in the virulence plasmid of *Salmonella enterica*. Front Microbiol 11:599931. 10.3389/fmicb.2020.599931. 33343541 PMC7746676

[22] Asano K, Mizobuchi K. 1998. Copy number control of IncIα plasmid ColIb-P9 by competition between pseudoknot formation and antisense RNA binding at a specific RNA site. EMBO J 17:5201–5213. 10.1093/emboj/17.17.5201. 9724656 PMC1170848

[23] Hiley L, Graham RMA, Jennison AV. 2019. Genetic characterisation of variants of the virulence plasmid, pSLT, in *Salmonella enterica* serovar Typhimurium provides evidence of a variety of evolutionary directions consistent with vertical rather than horizontal transmission. PLoS One 14:e0215207. 10.1371/journal.pone.0215207. 30973933 PMC6459517

[24] Stecher B, Denzler R, Maier L, Bernet F, Sanders MJ, Pickard DJ, Barthel M, Westendorf AM, Krogfelt KA, Walker AW, Ackermann M, Dobrindt U, Thomson NR, Hardt W-D. 2012. Gut inflammation can boost horizontal gene transfer between pathogenic and commensal Enterobacteriaceae. Proc Natl Acad Sci USA 109:1269–1274. 10.1073/pnas.1113246109. 22232693 PMC3268327

[25] Scherzinger E, Bagdasarian MM, Scholz P, Lurz R, Rückert B, Bagdasarian M. 1984. Replication of the broad host range plasmid RSF1010—requirement for three plasmid-encoded proteins. Proc Natl Acad Sci USA 81:654–658. 10.1073/pnas.81.3.654. 6322159 PMC344893

[26] Bakkeren E, Herter JA, Huisman JS, Steiger Y, Gül E, Newson JPM, Brachmann AO, Piel J, Regoes R, Bonhoeffer S, Diard M, Hardt W-D. 2021. Pathogen invasion-dependent tissue reservoirs and plasmid-encoded antibiotic degradation boost plasmid spread in the gut. eLife 10:e69744. 10.7554/eLife.69744. 34872631 PMC8651294

[27] Nair DVT, Venkitanarayanan K, Kollanoor Johny A. 2018. Antibiotic-resistant *Salmonella* in the food supply and the potential role of antibiotic alternatives for control. Foods 7:167. 10.3390/foods7100167. 30314348 PMC6210005

[28] Naylor NR, Atun R, Zhu N, Kulasabanathan K, Silva S, Chatterjee A, Knight GM, Robotham JV. 2018. Estimating the burden of antimicrobial resistance: a systematic literature review. Antimicrob Resist Infect Control 7:58. 10.1186/s13756-018-0336-y. 29713465 PMC5918775

[29] Jajere SM. 2019. A review of *Salmonella enterica* with particular focus on the pathogenicity and virulence factors, host specificity and antimicrobial resistance including multidrug resistance. Vet World 12:504–521. 10.14202/vetworld.2019.504-521. 31190705 PMC6515828

[30] Gormley EP, Davies J. 1991. Transfer of plasmid RSF1010 by conjugation from *Escherichia coli* to *Streptomyces lividans* and *Mycobacterium smegmatis*. J Bacteriol 173:6705–6708. 10.1128/jb.173.21.6705-6708.1991. 1657866 PMC209018

[31] De Gelder L, Ponciano JM, Joyce P, Top EM. 2007. Stability of a promiscuous plasmid in different hosts: no guarantee for a long-term relationship. Microbiology (Reading) 153:452–463. 10.1099/mic.0.2006/001784-0. 17259616

[32] Foley SL, Kaldhone PR, Ricke SC, Han J. 2021. Incompatibility group I1 (IncI1) plasmids: their genetics, biology, and public health relevance. Microbiol Mol Biol Rev 85:e00031-20. 10.1128/MMBR.00031-20. 33910982 PMC8139525

[33] Barthel M, Hapfelmeier S, Quintanilla-Martínez L, Kremer M, Rohde M, Hogardt M, Pfeffer K, Rüssmann H, Hardt W-D. 2003. Pretreatment of mice with streptomycin provides a *Salmonella enterica* serovar Typhimurium colitis model that allows analysis of both pathogen and host. Infect Immun 71:2839–2858. 10.1128/IAI.71.5.2839-2858.2003. 12704158 PMC153285

[34] Monack DM, Mueller A, Falkow S. 2004. Persistent bacterial infections: the interface of the pathogen and the host immune system. Nat Rev Microbiol 2:747–765. 10.1038/nrmicro955. 15372085

[35] Lawley TD, Chan K, Thompson LJ, Kim CC, Govoni GR, Monack DM. 2006. Genome-wide screen for *Salmonella* genes required for long-term systemic infection of the mouse. PLoS Pathog 2:e11. 10.1371/journal.ppat.0020011. 16518469 PMC1383486

[36] Kaiser P, Regoes RR, Dolowschiak T, Wotzka SY, Lengefeld J, Slack E, Grant AJ, Ackermann M, Hardt W-D. 2014. Cecum lymph node dendritic cells harbor slow-growing bacteria phenotypically tolerant to antibiotic treatment. PLoS Biol 12:e1001793. 10.1371/journal.pbio.1001793. 24558351 PMC3928039

[37] Stecher B, Paesold G, Barthel M, Kremer M, Jantsch J, Stallmach T, Heikenwalder M, Hardt W-D. 2006. Chronic *Salmonella enterica* serovar Typhimurium-induced colitis and cholangitis in streptomycin-pretreated Nramp1^+/+^ mice. Infect Immun 74:5047–5057. 10.1128/IAI.00072-06. 16926396 PMC1594839

[38] Claudi B, Spröte P, Chirkova A, Personnic N, Zankl J, Schürmann N, Schmidt A, Bumann D. 2014. Phenotypic variation of *Salmonella* in host tissues delays eradication by antimicrobial chemotherapy. Cell 158:722–733. 10.1016/j.cell.2014.06.045. 25126781

[39] Fisher RA, Gollan B, Helaine S. 2017. Persistent bacterial infections and persister cells. Nat Rev Microbiol 15:453–464. 10.1038/nrmicro.2017.42. 28529326

[40] von Wintersdorff CJH, Penders J, van Niekerk JM, Mills ND, Majumder S, van Alphen LB, Savelkoul PHM, Wolffs PFG. 2016. Dissemination of antimicrobial resistance in microbial ecosystems through horizontal gene transfer. Front Microbiol 7:173. 10.3389/fmicb.2016.00173. 26925045 PMC4759269

[41] Virolle C, Goldlust K, Djermoun S, Bigot S, Lesterlin C. 2020. Plasmid transfer by conjugation in Gram-negative bacteria: from the cellular to the community level. Genes (Basel) 11:1239. 10.3390/genes11111239. 33105635 PMC7690428

[42] Norman A, Hansen LH, Sorensen SJ. 2009. Conjugative plasmids: vessels of the communal gene pool. Philos Trans R Soc Lond B Biol Sci 364:2275–2289. 10.1098/rstb.2009.0037. 19571247 PMC2873005

[43] Garcia-Quintanilla M, Casadesus J. 2011. Virulence plasmid interchange between strains ATCC 14028, LT2, and SL1344 of *Salmonella enterica* serovar Typhimurium. Plasmid 65:169–175. 10.1016/j.plasmid.2010.12.001. 21145349

[44] Meyer R. 2009. Replication and conjugative mobilization of broad host-range IncQ plasmids. Plasmid 62:57–70. 10.1016/j.plasmid.2009.05.001. 19465049 PMC2752045

[45] Tietze E, Tschäpe H, Voigt W. 1989. Characterization of new resistance plasmids belonging to incompatibility group *IncQ*. J Basic Microbiol 29:695–706. 10.1002/jobm.3620291013. 2698955

[46] Sundin GW, Bender CL. 1996. Dissemination of the *strA-strB* streptomycin-resistance genes among commensal and pathogenic bacteria from humans, animals, and plants. Mol Ecol 5:133–143. 10.1111/j.1365-294x.1996.tb00299.x. 9147689

[47] van Overbeek LS, Wellington EMH, Egan S, Smalla K, Heuer H, Collard J-M, Guillaume G, Karagouni AD, Nikolakopoulou TL, van Elsas JD. 2002. Prevalence of streptomycin-resistance genes in bacterial populations in European habitats. FEMS Microbiol Ecol 42:277–288. 10.1111/j.1574-6941.2002.tb01018.x. 19709288

[48] Okoro CK, Kingsley RA, Quail MA, Kankwatira AM, Feasey NA, Parkhill J, Dougan G, Gordon MA. 2012. High-resolution single nucleotide polymorphism analysis distinguishes recrudescence and reinfection in recurrent invasive nontyphoidal *Salmonella typhimurium* disease. Clin Infect Dis 54:955–963. 10.1093/cid/cir1032. 22318974 PMC3297646

[49] Spees AM, Lopez CA, Kingsbury DD, Winter SE, Baumler AJ. 2013. Colonization resistance: battle of the bugs or menage a trois with the host? PLoS Pathog 9:e1003730. 10.1371/journal.ppat.1003730. 24278012 PMC3836981

[50] Brown K, DeCoffe D, Molcan E, Gibson DL. 2012. Diet-induced dysbiosis of the intestinal microbiota and the effects on immunity and disease. Nutrients 4:1095–1119. 10.3390/nu4081095. 23016134 PMC3448089

[51] De Palma G, Collins SM, Bercik P, Verdu EF. 2014. The microbiota-gut-brain axis in gastrointestinal disorders: stressed bugs, stressed brain or both? J Physiol 592:2989–2997. 10.1113/jphysiol.2014.273995. 24756641 PMC4214655

[52] Francino MP. 2015. Antibiotics and the human gut microbiome: dysbioses and accumulation of resistances. Front Microbiol 6:1543. 10.3389/fmicb.2015.01543. 26793178 PMC4709861

[53] Wilkins LJ, Monga M, Miller AW. 2019. Defining dysbiosis for a cluster of chronic diseases. Sci Rep 9:12918. 10.1038/s41598-019-49452-y. 31501492 PMC6733864

[54] Datsenko KA, Wanner BL. 2000. One-step inactivation of chromosomal genes in *Escherichia coli* K-12 using PCR products. Proc Natl Acad Sci USA 97:6640–6645. 10.1073/pnas.120163297. 10829079 PMC18686

[55] Jolley KA, Bray JE, Maiden MCJ. 2018. Open-access bacterial population genomics: BIGSdb software, the PubMLST.org website and their applications. Wellcome Open Res 3:124. 10.12688/wellcomeopenres.14826.1. 30345391 PMC6192448

[56] Bai Y, Müller DB, Srinivas G, Garrido-Oter R, Potthoff E, Rott M, Dombrowski N, Münch PC, Spaepen S, Remus-Emsermann M, Hüttel B, McHardy AC, Vorholt JA, Schulze-Lefert P. 2015. Functional overlap of the *Arabidopsis* leaf and root microbiota. Nature 528:364–369. 10.1038/nature16192. 26633631

[57] Zhang Z, Schwartz S, Wagner L, Miller W. 2000. A greedy algorithm for aligning DNA sequences. J Comput Biol 7:203–214. 10.1089/10665270050081478. 10890397

[58] Waterman SR, Holden DW. 2003. Functions and effectors of the *Salmonella* pathogenicity island 2 type III secretion system. Cell Microbiol 5:501–511. 10.1046/j.1462-5822.2003.00294.x. 12864810

[59] Lam LH, Monack DM. 2014. Intraspecies competition for niches in the distal gut dictate transmission during persistent *Salmonella* infection. PLoS Pathog 10:e1004527. 10.1371/journal.ppat.1004527. 25474319 PMC4256465

[60] Cunrath O, Bumann D. 2019. Host resistance factor SLC11A1 restricts *Salmonella* growth through magnesium deprivation. Science 366:995–999. 10.1126/science.aax7898. 31753999

