# Supplementary material for: The Mobilizable Plasmid P3 of Salmonella enterica Serovar Typhimurium SL1344 Depends on the P2 Plasmid for Conjugative Transfer into a Broad Range of Bacteria In Vitro and In Vivo
Source: J Bacteriol. 2022 Nov 16;204(12):e00347-22. doi: 10.1128/jb.00347-22 (PMC9765291; doi:10.1128/jb.00347-22)
Supplement: Supplemental file 1 — Fig. S1 to S6 and Tables S1 to S3. Download jb.00347-22-s0001.pdf, PDF file, 0.9 MB [file jb.00347-22-s0001.pdf]

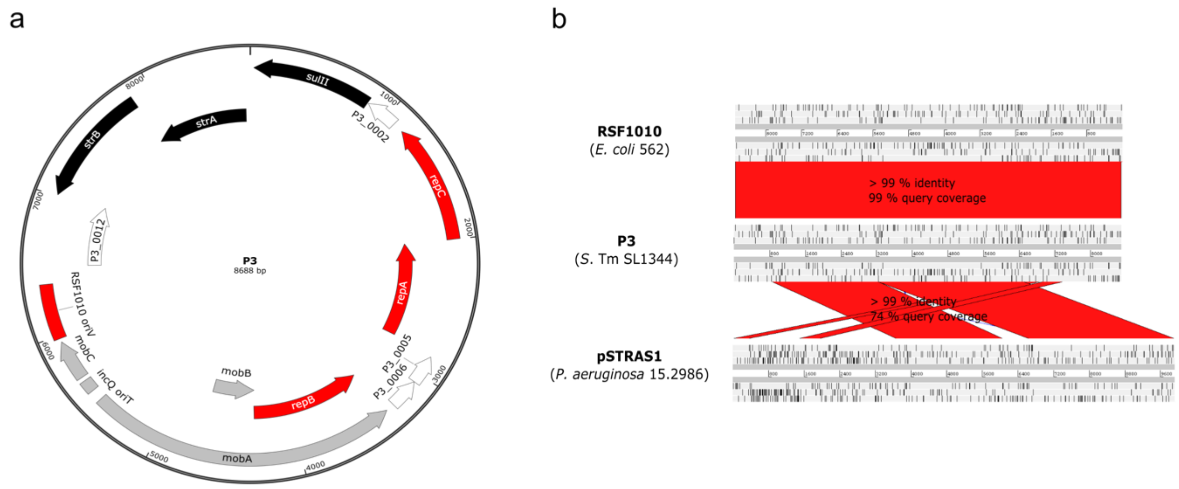

Fig S1: a | Genomic organization of the *S*. Tm SL1344 derived plasmid P3. Genes related to antibiotic resistance (black, *strAB*: streptomycin resistance, *sulII*: sulfonamide resistance), replication (red) and mobilization (grey) encoded on the plasmid P3 are depicted also indicating their orientation on the plasmid. Origin of transfer (*oriT*) and origin of replication (*oriV*) are indicated as boxed in respective colors. Genes indicated by white arrows have not been characterized so far. GenBank accession number: HE654726.1. Image was created using SnapGene software (from Insightful Science; available at snapgene.com). **b | Alignments.** Alignments to the originally isolated RSF1010 plasmid from *E. coli* strain 562 (GenBank accession number: M28229.1) and pSTRAS1 isolated from *P. aeruginosa* strain 15.2986 (GenBank accession number: MH463250.1) show the broad host range of RSF1010 plasmids, using the Artemis comparison tool (https://www.sanger.ac.uk/science/tools/artemiscomparison-tool-act). Red fill indicates high sequence identity (> 85% sequence identity).

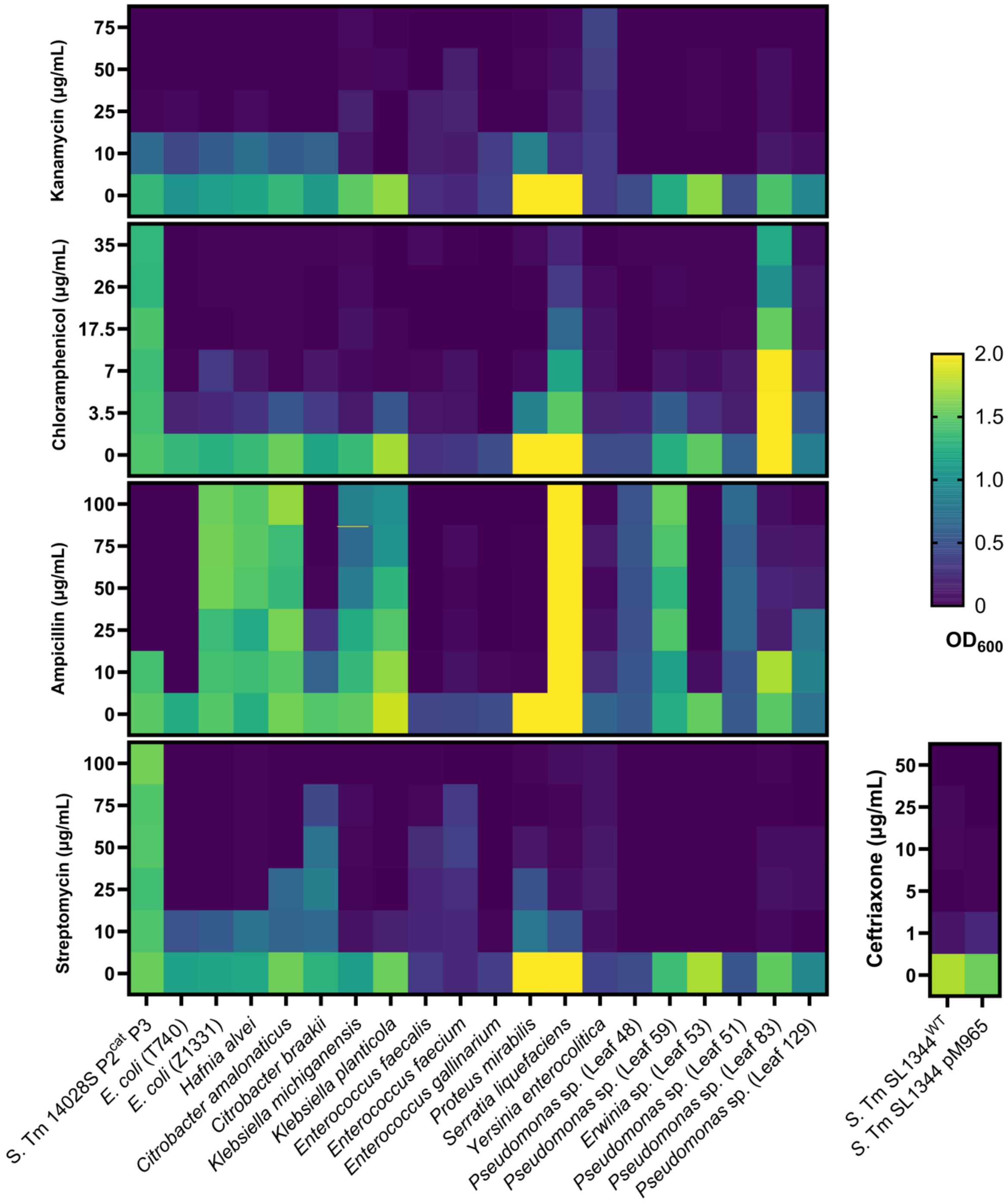

Figure S2: Heatmap of MIC assays. The heatmap shows OD_{600} values of 1 mL LB cultures that were inoculated with 1 x 10^7 CFUs of the different strains and incubated for 16 h at 30 °C / 37 °C with a range of depicted concentrations of kanamycin, chloramphenicol, ampicillin, streptomycin or ceftriaxone.

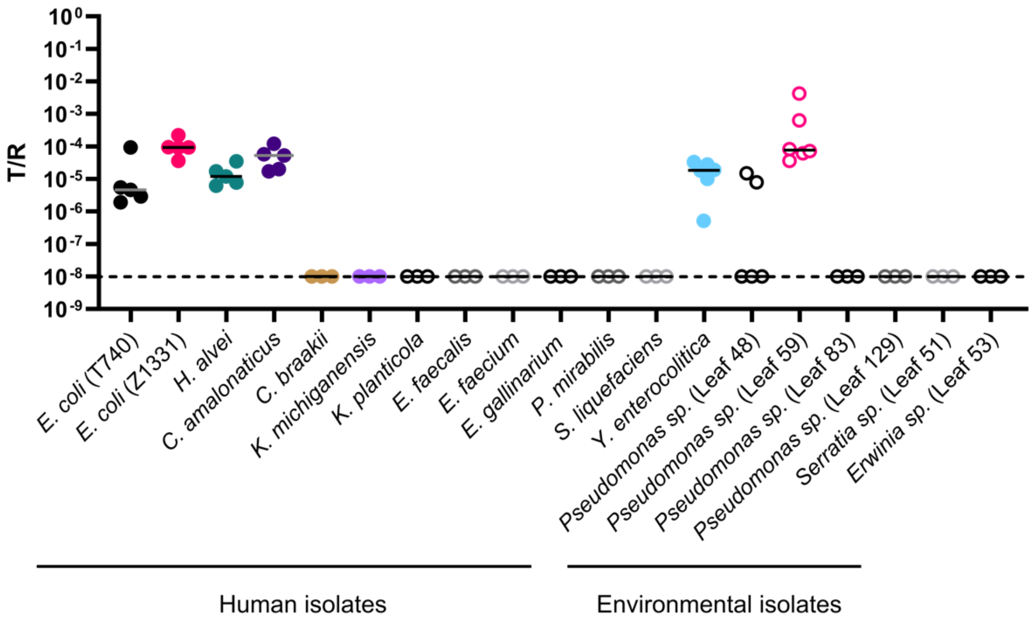

Fig S3: P3 can be transferred to various recipient strains in liquid cultures. Ratio$_{(R/T)}$ after overnight liquid mating for all recipient strains are shown. 2 x10^{7} CFUs of the recipients + *S*. Tm SL1344 (1:1 mix) were incubated in 5 mL LB overnight at 30 °C / 37 °C and donor, recipient and transconjugant counts were enumerated by respective plating. T/R: CFUs (transconjugant)/CFUs (recipient), n = 3 - 6 per group, LOD = 10^{-8}

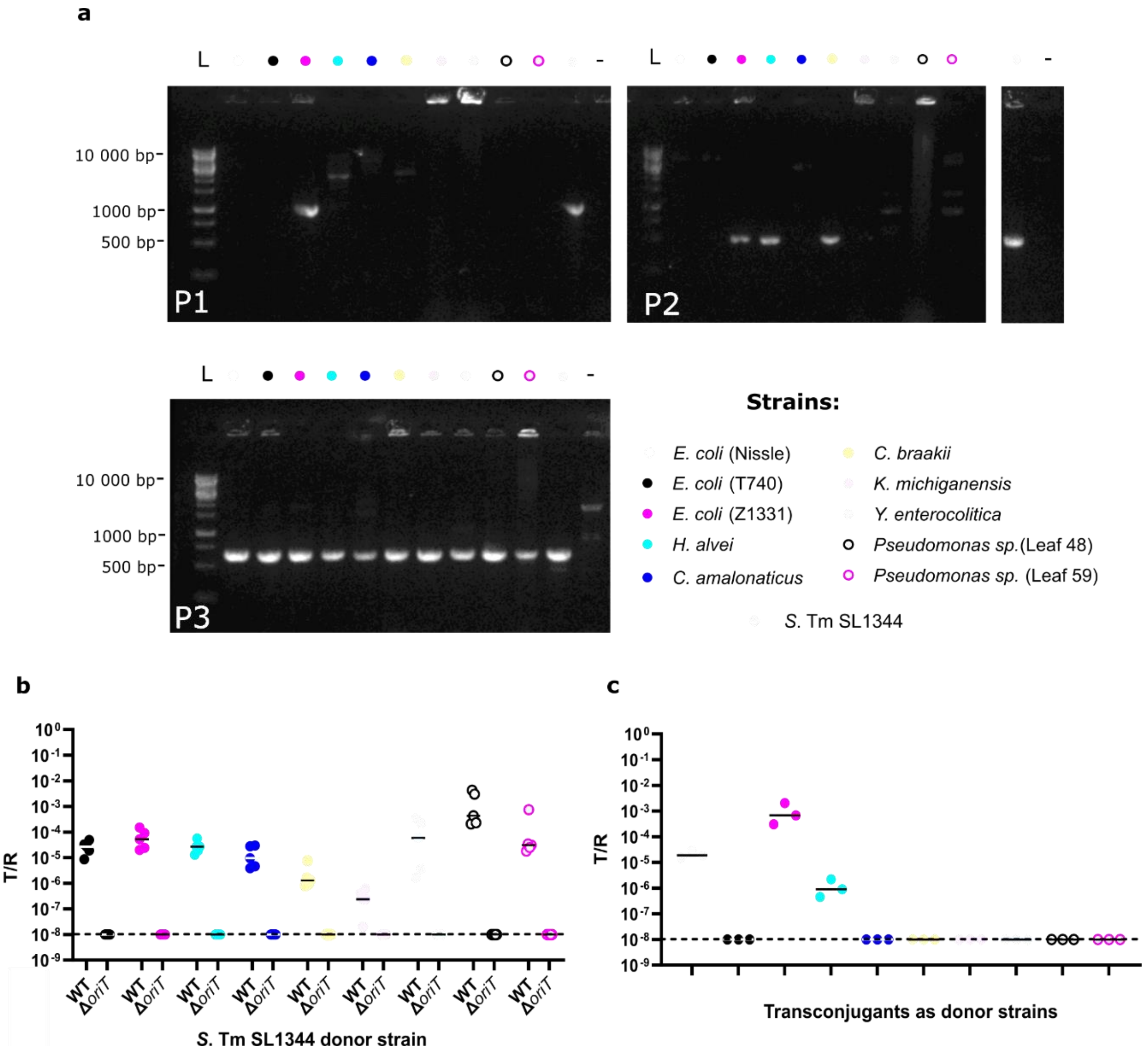

Fig S4: a | PCR verification of transconjugants. Transconjugants were verified by performing colony PCR. P1 should yield a PCR product of 600 bp, P2 a PCR product of 500 bp and P3, a PCR product of 700 bp. P3, but not P1 or P2, was detected in all transconjugant colonies tested from the experiment shown in **Fig. 1B**. **b | P3 is transferred by conjugation in all recipients.** Ratio$_{T/R}$ of overnight surface mating with *S*. Tm SL1344 P3$^{\Delta oriT}$ or *S.* Tm SL1344^{WT} and depicted recipient strains. 1:1 mix incubated on LB agar overnight at 30 °C / 37 °C depending on the recipient strain. Donor, recipient and transconjugant counts were enumerated by respective plating, n = 5. **c | P3 is further transferred by donors additionally harboring P2.** Ratio$_{R/T}$ of overnight surface mating with *E. coli* W3110 *tsr::kmR* as recipient and depicted transconjugant strains as donors. Same experimental setup as described in a, n = 3. LOD = 10^{-8}.

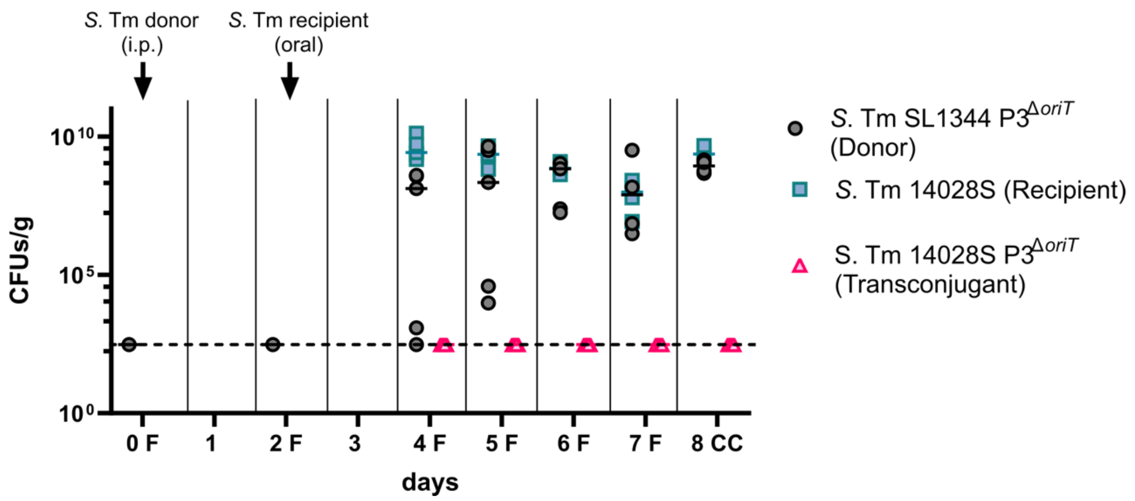

Fig S5: P3 transmission in the gut lumen is dependent on conjugative transfer. Total CFU counts of donor, recipient and transconjugant populations in the feces and cecal contents are depicted. Mice were infected with 5 x 10^7 CFUs of the recipient on day 0 (i. p.) and with 5 x 10^7 CFUs of the donor *S*. Tm SL1344 WITS-1-*kmR* on day 2 (oral). Donors, recipients and transconjugants are depicted as grey circles, cyan squares and pink triangles, respectively. F: fecal sample, CC: cecal content. n = 4.

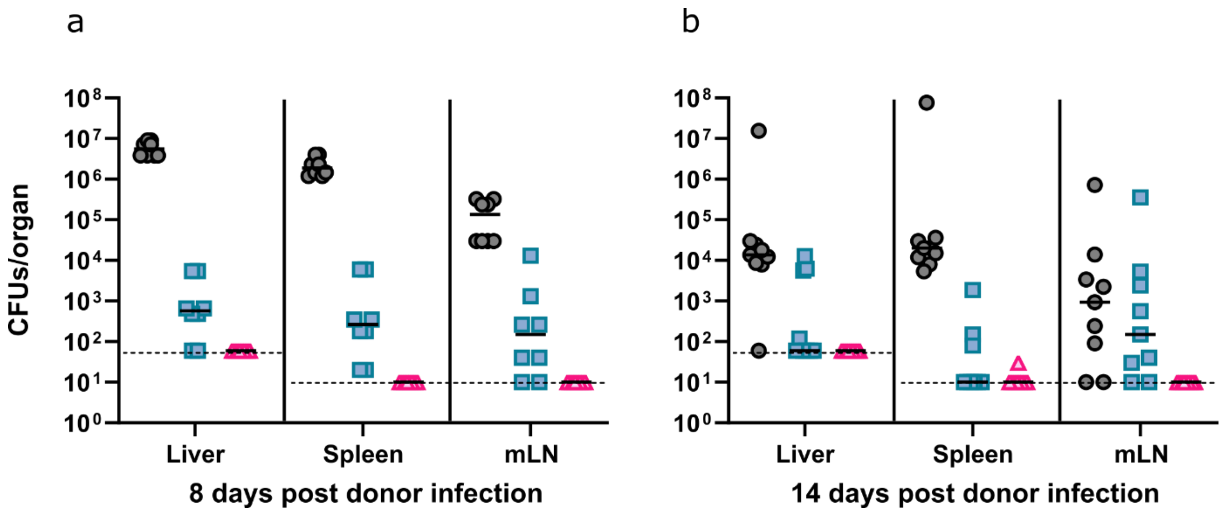

Fig S6: Total CFU counts for systemic *S.* Tm infection. a | Total CFU counts for reseeding donor experiments (Fig 3a-c). Donor (grey circles), recipient (cyan squares) and transconjugant (pink triangles) populations in the liver, spleen and mesenteric lymph nodes (mLN) are depicted. Mice were infected with 5 x 10^7 CFUs of the donor *S.* Tm SL1344 (Amp^R, Km^R) on day 0 (i. p.) and with 5 x 10^7 CFUs of the recipient *S*. Tm 14028S (Amp^R, Cm^R) on day 2 (oral). n = 9, two independent experiments. **b | Total CFU counts for systemic organs of persister experiments (Fig 3d-f).** Donor, recipient and transconjugant populations in the liver, spleen and mesenteric lymph nodes are depicted. Mice were infected with 5 x 10^7 CFUs of the donor *S.* Tm SL1344 (Amp^R, Km^R) on day 0 (i. p.) and with 5 x 10^7 CFUs of the recipient *S*. Tm 14028S (Amp^R, Cm^R) on day 7 (oral). n = 9, two independent experiments.

Table S1: Bacterial strains used in this study.

Strain ID	Strain	Genotype	Marker	T [°C]	Experiment	Reference
Z1629	*S.*Tm SL1344	SB300	Sm50, lac^{-}	37	Donor for *in vitro* matings and *in vivo* interspecific transfer exp.	Hardt lab strain collection, (1, 2)
Z7740	*E. coli* Z1331	*E. coli* Z1331 *yidX*-stop-WITS1-AmpR	Amp100, lac^{+}	37	Recipient screen, human isolate	Hardt lab strain collection
YE	*Yersinia enterocolitica* 245	Wild type	Km50	30	Recipient screen	Hardt lab strain collection
KPl	*Klebsiella planticola* 4481	Wild type	Amp100, lac^{+}	37	Recipient screen	Hardt lab strain collection
SL	*Serratia liquefaciens*	Wild type	Amp100	30	Recipient screen	Hardt lab strain collection
Leaf48	*Pseudomonas sp.* Leaf48	Wild type	Amp100	30	Recipient screen, Environm. Isolate	(3)
Leaf59	*Pseudomonas sp.* Leaf59	Wild type	Cm25, Amp100	30	Recipient screen, Environm. Isolate	(3)
Leaf83	*Pseudomonas sp.* Leaf83	Wild type	Cm35, Sm50	30	Recipient screen, Environm. Isolate	(3)
Leaf129	*Pseudomonas sp.* Leaf129	Wild type		30	Recipient screen, Environm. Isolate	(3)
Leaf51	*Pseudomonas sp.* Leaf51	Wild type	Amp100	30	Recipient screen, Environm. Isolate	(3)
Leaf53	*Erwinia sp.* Leaf53	Wild type		30	Recipient screen, Environm. Isolate	(3)
Z6026	*Hafnia alvei*	Wild type		37	Recipient screen, human isolate	Hardt lab strain collection
T740	*E. coli*	Wild type	lac^{+}	37	Recipient screen, human isolate	Hardt lab strain collection
T706	*Citrobacter braakii*	Wild type	lac^{+}	37	Recipient screen, human isolate	Hardt lab strain collection
T707	*Enterococcus faecium*	Wild type	lac^{+}	37	Recipient screen, human isolate	Hardt lab strain collection
T727	*Enterococcus gallinarium*	Wild type	lac^{+}	37	Recipient screen, human isolate	Hardt lab strain collection
T737	*Klebsiella michiganensis*	Wild type	Amp100, lac^{+}	37	Recipient screen, human isolate	Hardt lab strain collection
T746	*Proteus mirabilis*	Wild type		37	Recipient screen, human isolate	Hardt lab strain collection
T747	*Citrobacter amalonaticus*	Wild type	Amp100	37	Recipient screen, human isolate	Hardt lab strain collection
T749	*Enterococcus faecalis*	Wild type	lac^{+}	37	Recipient screen, human isolate	Hardt lab strain collection
T213	*S.* Tm SB300	*S.* Tm SB300 WITS1-*kmR*	Sm50, Km50	37	Donor for *in vivo* reseeding and persister exp.	Hardt lab strain collection
M3145	*S.* Tm 14028S	*S.* Tm 14028S *marT::cat*	Cm35	37	Recipient for *in vitro* and *in vivo* exp.	Hardt lab strain collection
Z7870	*S.* Tm SB300	*S.* Tm SL1344 $P3^{\Delta oriT}$ (*oriT::kmR*)	Sm50, Km50	37	Donor for *in vitro* and *in vivo* exp.	This study
Z1852	*S.* Tm ATCC 14028S	*S.* Tm 14028S $P2^{cat}$	Cm35	37	Recipient for *in vitro* experiments	(4)
Z2287	*S.* Tm SB300	*S.* Tm SB300 ΔP2 pM975	Sm50, Amp100	37	Donor for *in vitro* experiments	(5)

Z7707	*E. coli* W3110	*tsr::kmR*	Km50	37	Recipient for *in vitro* experiments	Hardt lab strain collection
Z7722	*E. coli* W3110	*E. coli* W3110 RpoS+ *tsr::kmR* pM965	Km50	37	Template strain for Kanamycin cassette	Hardt lab strain collection

Amp100: resistant to 100 µg/mL of ampicillin, Sm: Streptomycin, Km: Kanamycin, Cm: Chloramphenicol, lac^{+}: Lactose fermenting

Table S2: Plasmids used in this study.

Plasmid	T [°C]	Description	Backbone	Reference
pM965	37	AmpR	pBR322	(6)
pACYC184	37	CmR	p15A	(7)
P3	37	SmR	pRSF1010	(8)
pKD46	30	λ-Red recombinase, expression induced by addition of arabinose; AmpR	pINT-ts	(9)

AmpR: ampicillin resistance, CmR: chloramphenicol resistance, SmR: streptomycin resistance, oriR: origin of replication

Table S3: Primers used in this study.

Primer	Sequence 5’ to 3’	Info
LeoL287	TGAACAGCCGCCCATTGGC	Confirmation of P3 uptake, 300 bp upstream of oriT
LeoL288	CTTCTGGCGTGGCGGCA	Confirmation of P3 uptake, 300 bp downstream of oriT
LeoL292	GTAAGGTGATAAATCGCCATGCTGCCTCGCTGTTGCTTTT<br>GCTTTTCGGCTCCATGCAATGGCCCTCGGAGAGCGCACC<br>GCCCGAAGGGTGGCCGTTAGGATTCCGGGGATCCGTCG<br>ACC	100 bp upstream P3 oriT + KmR fwd primer, used for oriT disruption
LeoL293	CATTGATTCGCGCTCGTTGTTCTTCGAGCTTGGCCAGCC<br>GATCCGCCGCCTTGTTGCTCCCCTTAACCATCTTGACAC<br>CCCATTGTTAATGTGCTGTCTCTGTAGGCTGGAGCTGCTT<br>CG	100 bp downstream P3 oriT + KmR rev primer,used for oriT disruption
LeoL300	GCATTTCAGTCA GTTGCTC	Confirmation of $P2^{cat}$, CmR cassette fwd primer
LeoL301	CGACATGGAAGC CATCA	Confirmation of $P2^{cat}$, CmR cassette rev primer
LeoL318	CCGCTCGAGCGGTGGCTCAGGCGGGA	$oriR^{P1}$ amplification fwd primer + XhoI restriction site

LeoL319	AAGGAAAAAAGCGGCCGCAAAAGGAAAACCTGAACCACTGGACATC	oriR^{P1} amplification rev primer + NotI restriction site
LeoL320	CATCATGCGCTCTTTCACGCG	oriR insert verification fwd primer
LeoL321	ACCAGAAAAGCAAAAACCCCGATA	oriR insert verification rev primer
LeoL342	GCTGGATAAATATCTTCTTTTTTTCCTGGCA	oriR verification
LeoL343	TTTAACAGGCTGACACTGACATACAGC	oriR verification

oriT: origin of transfer, oriR: origin of replication, fwd: forward, rev: reverse, KmR: kanamycin resistance, CmR: chloramphenicol resistance

SUPPLEMENTARY REFERENCES

1. Kroger C, Dillon SC, Cameron AD, Papenfort K, Sivasankaran SK, Hokamp K, Chao Y, Sittka A, Hebrard M, Handler K, Colgan A, Leekitcharoenphon P, Langridge GC, Lohan AJ, Loftus B, Lucchini S, Ussery DW, Dorman CJ, Thomson NR, Vogel J, Hinton JC. 2012. The transcriptional landscape and small RNAs of *Salmonella enterica* serovar Typhimurium. Proc Natl Acad Sci U S A 109:E1277-E1286.
2. Hoiseth SK, Stocker BAD. 1981. Aromatic-dependent *Salmonella* Typhimurium are non-virulent and effective as live vaccines. Nature 291:238-239.
3. Bai Y, Muller DB, Srinivas G, Garrido-Oter R, Potthoff E, Rott M, Dombrowski N, Munch PC, Spaepen S, Remus-Emsermann M, Huttel B, McHardy AC, Vorholt JA, Schulze-Lefert P. 2015. Functional overlap of the *Arabidopsis* leaf and root microbiota. Nature 528:364-369.
4. Stecher B, Denzler R, Maier L, Bernet F, Sanders MJ, Pickard DJ, Barthel M, Westendorf AM, Krogfelt KA, Walker AW, Ackermann M, Dobrindt U, Thomson NR, Hardt WD. 2012. Gut inflammation can boost horizontal gene transfer between pathogenic and commensal Enterobacteriaceae. Proc Natl Acad Sci U S A 109:1269-1274.
5. Bakkeren E, Huisman JS, Fattinger SA, Hausmann A, Furter M, Egli A, Slack E, Sellin ME, Bonhoeffer S, Regoes RR, Diard M, Hardt WD. 2019. *Salmonella* persisters promote the spread of antibiotic resistance plasmids in the gut. Nature 573:276-280.
6. Stecher Br, Hapfelmeier S, Müller C, Kremer M, Stallmach T, Hardt W-D. 2004. Flagella andchemotaxis are required for efficient induction of *Salmonella enterica* serovar Typhimurium colitis in streptomycin-pretreated mice. Infection and Immunity 72:4138-4150.
7. Chang ACY, Cohen SN. 1978. Construction and characterization of amplifiable multicopy DNA cloning vehicles derived from the P15A cryptic miniplasmid. Journal of Bacteriology 134:1141-1156.
8. Scholz P, Haring V, Wittmann-Liebold B, Ashman K, Bagdasarian M, Scherzinger E. 1989. Complete nucleotide sequence and gene organization of the broad-host-range plasmid RSF1010. Gene 75:271-288.
9. Datsenko KA, Wanner BL. 2000. One-step inactivation of chromosomal genes in *Escherichia coli* K-12 using PCR products. Proceedings of the National Academy of Sciences 97:6640-6645.
